# Imposter Phenomenon and Its Predictors in Saudi ENT Specialists: A Cross-Sectional Study

**DOI:** 10.7759/cureus.54918

**Published:** 2024-02-26

**Authors:** Abdulaziz M Alrasheed, Khalid S Alomar, Ahmed Al Olaywi

**Affiliations:** 1 Family and Community Medicine, King Saud University Medical City, Riyadh, SAU; 2 Otolaryngology - Head and Neck Surgery, King Saud University Medical City, Riyadh, SAU; 3 Otolaryngology - Head and Neck Surgery, King Abdulaziz University Hospital, Riyadh, SAU; 4 Otolaryngology - Head and Neck Surgery, Security Forces Hospital, Riyadh, SAU

**Keywords:** saudi arabia, otolaryngologists, ent, impostor phenomenon, impostor syndrome

## Abstract

Background

Impostor syndrome (IS) is characterized by an ongoing disbelief in the authenticity of one's accomplishments, attributing success to luck rather than to one's own ability or hard work. The syndrome has detrimental consequences in both personal and career aspects and is closely linked to emotional exhaustion, stress, and depressive symptoms. This study aimed to determine the prevalence of IS among otolaryngologists practicing in Saudi Arabia.

Methods

We performed a cross-sectional survey from August to October 2022 that targeted both practicing otolaryngologists and those still in their training phase in Saudi Arabia. The survey, delivered via email, tapped into the Saudi Commission for Health Specialties (SCFHS) database to reach all registered otolaryngologists. The survey tool comprised questions on demographic details and employed the Clance Impostor Phenomenon (IP) Scale to evaluate the presence of IS.

Results

Out of 80 respondents, males (n = 46) were 57.5%, and 18.8% were consultants (n =15). The study found a 27.5% prevalence rate of IS among the surveyed otolaryngologists (n =80), with a mean score of 56.79 ± 12.98. In terms of severity, 62.5% (n = 50) had a moderate level of IS, 25.0% (n = 20) had high IS, and 5.0% (n = 4) had intense IS. It was significantly more common with resident otolaryngologists as compared to consultants (*X^2 ^*= 7.476, *df* = 3; *p* = 0.048), but there was no significant association in terms of gender (*X^2^*=3.418, *df* =1; *p* = 0.064), type of hospital (*X^2^*= 6.351, *df* = 3; *p* = 0.096), or fellowship subspecialty (*X^2^*= 2.291, *df* = 4; *p* = 0.681).

Conclusions

The study detected that 36.9% of otolaryngologists (n = 17) experienced IS, with trainees being more susceptible than consultants and fellows. Further investigations to explore the scope and underlying reasons are recommended.

## Introduction

Impostor syndrome (IS) is characterized by a persistent inability to attribute one's achievements to personal competence, instead ascribing success to luck or other external factors [1-2]. This self-doubt often manifests as a sense of being a fraud, which is known as impostorism [3]. Through the lens of Bandura's self-efficacy theory, IS can severely impact an individual's self-confidence and potential for success [4-6]. Given that low self-efficacy is known to affect a range of life outcomes, from academic achievement to mental well-being, it becomes imperative to delve deeper into the complexities of IS [7].

Contrary to early beliefs that IS was limited to specific groups (e.g., women), more recent research indicates that it is pervasive across genders, diverse cultures, and various professional fields [8-9]. Interestingly, up to 70% of people, including high-achievers, report experiencing elements of impostorism during their careers [10]. While IS can sometimes fuel a greater ambition to excel, leading to career advancement, it also has its downsides. Studies have revealed that IS is correlated with emotional fatigue, heightened work-family conflicts, and decreased job satisfaction [11-12]. Additionally, individuals with IS are more prone to psychological distress, such as depression, low self-esteem, and anxiety, when faced with failure. Notably, IS is also associated with perfectionistic tendencies and elevated stress levels at work, and there is emerging evidence linking it to both professional burnout and suicidal ideation [13].

Within the field of medicine, the understanding of IS remains limited, posing a significant issue given the profession's high-stress environment and demands for perfectionism. Recent data show that a substantial percentage of physicians and medical residents are experiencing burnout, a condition to which IS contributes [14]. Therefore, understanding the prevalence and impact of IS within the medical community is crucial, especially considering its significant role in the rising levels of burnout among healthcare professionals. In this study, we aimed to assess the prevalence and severity of IS among licensed Ear, Nose, and Throat (ENT) residents, fellows, and consultants in Saudi Arabia.

## Materials and methods

Study design and settings

This cross-sectional study was performed between January and July 2023. The study was national in scope, targeting all ENT residents, fellows, and consultants who were licensed by the Saudi Commission for Health Specialties (SCFHS) across Saudi Arabia. The study protocol received ethical approval from the King Saud University Institutional Review Board (E-23-7612). A written consent was obtained from all participants prior.

Participants

The inclusion criteria comprised ENT residents, fellows, and consultants currently registered with SCFHS. Exclusion criteria were professionals from other medical specialties, interns, and medical students.

Variables

Socio-demographic characteristics, including gender and level of training, were collected. The primary outcome variable was the incidence of IS, measured using the Clance Impostor Phenomenon (IP) Scale [15].

Data sources/measurement

An online questionnaire was distributed via personal email addresses to the eligible participants. The Clance IP Scale is a validated survey consisting of 20 questions on a 5-point scale to assess the characteristics and severity of the IP [15]. The questionnaire was administered through an online platform called SurveyMonkey® (SurveyMonkey, San Mateo, CA).

Statistical analysis

All statistical procedures were carried out using the SPSS software (Statistical Packages for Social Sciences), version 26, developed by IBM Corp., based in Armonk, NY, USA. For categorical variables, descriptive statistics were presented as frequencies and percentages, while continuous variables were summarized using the mean and standard deviation (SD). Chi-square tests were used to investigate the relationship between IS and various socio-demographic characteristics among urologists. A P-value of ≤ 0.05 was considered statistically significant.

## Results

Socio-demographic characteristics

In this study involving 80 otolaryngologists, male respondents (n = 46, 57.5%) were more than females (n = 34, 42.5%). Most participants worked in the Central Region (n = 62, 77.5%). However, a representation from the Western (n = 7, 8.8%), Northern (n = 3, 3.8%), Eastern (n =7, 8.8%), and Southern regions (n = 1, 1.3%) was also included. Regarding the level of working experience, the majority of participants were residents (n = 46, 57.5%), followed by consultants (n = 15, 18.8%), fellows (n = 10, 12.5%), and registrars (n = 9, 11.3%).

In terms of the type of hospital, most were working in the Ministry of Health Hospitals (n = 28, 35%), followed by the Military Hospitals (n = 24, 30%), Academic/University Hospitals (n = 18, 22.5%), and Private Hospitals (n = 10, 12.5%). Regarding specialty, the majority were general otolaryngologists (n = 59, 73.3%), while the remaining were among various specialties, including head and neck surgery (n = 7, 8.8%), facial plastic surgery (n = 5, 6.3%), and paediatric otolaryngology (n = 3, 3.8%). The otology, neurotology, lateral skull base unit, laryngology and phonosurgery, and rhinology and skull base surgery were summed up as others (n = 6, 7.5%), as shown in Table 1.

**Table 1:** Socio-demographic characteristics of respondents (n=80). Variable with multiple response answers. Data are presented as N (%).

Variables	n (%)
Gender	
Male	46 (57.5)
Female	34 (42.5)
Region of work	
Central region	62 (77.5)
Eastern region	7 (8.8)
Western region	7 (8.8)
Northern region	3 (3.8)
Southern region	1 (1.3)
Level of working experience	
Resident	46 (57.5)
Fellows	10 (12.5)
Registrar	9 (11.3)
Consultant	15 (18.8)
Type of hospital	
Ministry of health hospitals	28 (35.0)
Military hospitals	24 (30.0)
University academic hospitals	18 (22.5)
Private hospitals	10 (12.5)
Fellowship specialty	
General otolaryngology	59 (73.8)
Head and neck	7 (8.8)
Fascial plastic surgery	5 (6.3)
Pediatric otolaryngology	3 (3.8)
Others	6 (7.5)

Prevalence and severity of imposter syndrome

The Imposter Syndrome (IS) score was calculated as (n =80, mean and SD = 56.79±12.98) with minimum and maximum scores (37.0; 100.0), respectively. In terms of the level of IS, 27.5% (n = 22) of the participants scored 62 or higher, falling into the "impostor" category, while the majority (n = 58, 72.5%) had scores below 62, indicating normal levels of impostor feelings. Regarding the severity of IS, 7.5% (n = 6) of the participants were categorized as experiencing mild symptoms (score ≤ 40), 62.5% (n =50) had moderate IS (score 40-60), 25.0% (n =20) had high IS (score 61-80), and 5.0% (n =4) had intense IS (score >80), as shown in Table 2.

**Table 2:** Prevalence of impostor phenomenon using Clance IP scale (n=80).

Impostor variables	N (%)
Impostor phenomenon score (mean ± SD)	50.81 ± 16.6
Level of impostor syndrome	
Impostor (score ≥ 62)	22 (27.5%)
Normal (score <62)	58 (72.5%)
The severity of impostor syndrome	
Mild (score ≤ 40)	24 (30%)
Moderate (score 40–60)	34 (42.5%)
High (score 61–80)	17 (21.3%)
Intense (score > 80)	5 (6.2%)

Factors associated with imposter syndrome

Chi-square statistics were used to examine the association between categorical variables. In examining the relationship between imposter syndrome (IS) and socio-demographic characteristics among otolaryngologists, a significant difference was observed only in the level of working experience (*X*^2^ = 7.476, df = 3, p = 0.048). Specifically, no consultants (0%) fell into the 'impostor' category, compared to residents (n = 17, 36.9%), fellows (n = 3, 30%), and registrars (n =2, 22.2%). Other socio-demographic variables such as gender (*X*^2^ =3.418, df = 1, p =.064), type of hospital (*X*^2^ =6.351, df = 3, p = 0.096), and fellowship subspecialty (*X*^2^ =2.291, df = 4, p = 0.681) did not show statistically significant relationships with the presence of IS (Table 3).

**Table 3:** Relationship between imposter syndrome and sociodemographic characteristics of respondents (n=80). *Significant at alpha level <0.05; **included neurotology, airway surgery, rhinology and skull base surgery, phono surgery, etc.

Variable	Imposter n (%)	Normal n (%)	Total (n = 80) %	X^2^	df	P- value
Gender						
Male	9 (19.6)	37 (80.4)	46 (100)	3.418	1	0.064
Female	13 (38.2)	21 (61.8)	34 (100)			
Level of work experience						
Resident	17 (36.9)	29 (63.1)	46 (100)	7.476	3	0.048*
Fellow	3 (30.0)	7 (70.0)	10 (100)			
Registrar	2 (22.2)	7 (77.8)	09 (100)			
Consultant	0 (0.00)	15 (100)	15 (100)			
Type of hospital						
Ministry of health hospitals	6 (21.4)	22 (78.6)	28 (100)	6.351	3	0.096
Military hospitals	5 (20.8)	19 (79.2)	24 (100)			
University hospitals	5 (27.8)	13 (72.2)	18 (100)			
Private hospitals	6 (60.0)	4 (40.0)	10 (100)			
Type of specialty						
General otolaryngology	17 (28.8)	42 (71.2)	59 (100)	2.291	4	0.681
Head and neck	1 (14.3)	6 (85.7)	7 (100)			
Fascial plastic	2 (40.0)	3 (60.0)	5 (100)			
Pediatric otolaryngology	0 (00.0)	3 (100.0)	3 (100)			
Others**	2 (33.3)	4 (66.7)	6 (100)			

## Discussion

In this cross-sectional study, our findings highlight that the prevalence of IS among otolaryngologists in Saudi Arabia is 27.5%. This prevalence is relatively lower than the figures reported in the literature. In the United States (US), Henning et al. [16], Villwock et al. [17], and Swope and Thompson [18] reported that 30.2%, 38%, and 50% of the medical students had clinically significant IS, respectively. Similarly, Oriel et al. showed that the prevalence of IS among US family medicine resident physicians was 33% [19]. In Canada and Malaysia, Legassie et al. [20] and Ikbaal and Salim Musa [21] also reported a higher prevalence in resident physicians at 43.8% and 45.7%, respectively. Additionally, two Pakistani studies reported a higher prevalence of IS in medical students (47.3% and 54.5%) [22,23]. Mascarenhas et al. showed that 41.3% of the Iranian resident physicians had confirmed IS [24]. These variations in prevalence rates suggest that regional or cultural disparities may be impacting the prevalence of IS within the medical community. Additionally, education and healthcare systems could influence these rates.

In our study of Saudi Arabian otolaryngologists, 42.5% had moderate IS, 21.3% had severe IS, and 6.2% had intense IS. An Indian study showed that 44.7% of participants had moderate IS levels, 47.3% had severe IS, and 5.3% had intense IS [24]. Although our findings in terms of moderate and intense IS aligned closely with their findings, the percentage of severe cases is quite lower in our study. Likewise, our study revealed a much lower percentage of severe cases compared to those reported in Pakistan (54.5%) [23]. In another Saudi study that was conducted on surgical and medical residents in Makkah, the percentages of moderate, high, and intense IS were 45.9%, 47.3%, and 2.9%, respectively [25].

The implications of impostor syndrome on medical professionals' self-esteem and performance are too significant to ignore, and the prevalence across varying settings and specialties suggests a systemic issue that warrants attention. Mascarenhas et al. highlighted that high levels of self-esteem and low levels of IS are conducive to efficient medical practice [24]. Given that IS is common among both surgical and medical residents, these observations highlight the need for more extensive research in diverse healthcare settings. Understanding how the work environment might contribute to varying levels of IS across age groups and specialties could offer valuable insights into the syndrome's prevalence and severity [15]. In addition, healthcare organizations, educational institutions, and governing bodies should prioritize the investigation and intervention of impostor syndrome, facilitating a healthier work environment and better patient outcomes [9].

In this study, we could not find a significant difference between males and females in terms of the prevalence of IS. This finding aligns with multiple reports indicating no significant gender disparities in the manifestation of IP characteristics [21,24,26,27]. Conversely, research by Villwock et al. [17] showed that IS was more common in women than men (49.4% vs. 23.7%; P = 0.004). Additionally, Legassie et al. [20], Oriel et al. [19], Henning et al. [16], Latif [22], and Almatrafi et al. [25] demonstrated that females were significantly more prone to impostor feelings compared to their male counterparts. A recent systematic review also suggested that women tend to report higher rates of impostor feelings, although this could be a byproduct of early IP research primarily focusing on women [9]. Despite these conflicting results, this observation suggests that both male and female healthcare professionals are susceptible to impostor feelings, prompting healthcare organizations to consider impostor syndrome as an issue affecting their entire workforce, not just women.

Our findings indicate a higher likelihood of resident otolaryngologists exhibiting characteristics of IS compared to consultants, registrars, and fellows. This observation closely aligns with the study by Leach et al., which found surgical residents to score noticeably higher on the Clance IP scale when compared to attending surgeons [27]. Although Almatrafi et al. [25] also noted an elevated sense of impostorism among surgical residents, especially those in the early years of their residency, their results did not achieve statistical significance (p > 0.05). Additionally, their study revealed that other variables had no meaningful impact on the IS scores among residents. Interestingly, the study by Villwock et al. indicated that one in four young undergraduate medical students suffers from IS, which was found to be strongly correlated with indices of burnout in this demographic [17]. Several studies have reported that medical students and residents with low self-esteem tend to report elevated levels of IS [21,24,26]. Moreover, perfectionistic tendencies have also been identified as a contributing factor to an increased risk of IS [16]. Existing literature further posits that the hierarchical structure inherent in medical education, coupled with the prevailing culture within the medical field, could exacerbate IS symptoms. Specifically, in such settings, seeking assistance or admitting a lack of knowledge are often viewed as signs of weakness [28,29].

Our study is subject to some limitations. The cross-sectional design limits our ability to establish causality. Our focus on otolaryngologists in Saudi Arabia also means that the findings may not be generalizable to other medical specialties or countries. Additionally, reliance on self-reported measures and the use of email invitations for study participation may have introduced some selection bias.

Future directions

The findings from our study offer significant implications for both research and clinical practice. While the prevalence of IS among otolaryngologists in Saudi Arabia is comparatively lower than in other countries, the impact on residents appears significant, aligning with the notion that early career medical professionals may be more susceptible. This suggests that early interventions aimed at identifying and managing IS during the formative years of medical training may offer the most impact. It also signifies the importance of developing standardized interventions that could be implemented in healthcare and educational settings. Furthermore, the absence of significant gender differences in IS prevalence in our study contrasts with some previous findings and could point to cultural differences affecting the expression of IS. This warrants a more nuanced, cross-cultural understanding of IS, allowing for more effective, culturally sensitive interventions. The relationship between IS and the level of training suggests that career progression may mitigate IS symptoms, offering a pathway for potential interventions that support career development and mentorship as a means of reducing IS.

## Conclusions

This study highlights the importance of understanding the prevalence and severity of IS among healthcare professionals, particularly in the Saudi context. It underscores the need for further research to investigate how cultural and professional contexts might influence IS and how early interventions can mitigate its negative consequences. Healthcare organizations and educational institutions should consider incorporating awareness and interventions for IS into their professional development programmes to enhance both the well-being of their staff and the quality of care provided to patients.
